# Outcomes of the Novel Supreme Drug-Eluting Stent in Complex Coronary Lesions: A PIONEER III Substudy

**DOI:** 10.1016/j.jscai.2021.100004

**Published:** 2022-01-30

**Authors:** Kush P. Patel, Alexandra J. Lansky, Dean J. Kereiakes, Stephan Windecker, Ecaterina Cristea, Cody Pietras, Ovidiu Dressler, M. Ozgu Issever, Michael Curtis, Barry Bertolet, James P. Zidar, Pieter C. Smits, Victor Alfonso Jiménez Díaz, Brent McLaurin, David A. Brogno, Luc Janssens, Mathias C. Vrolix, Iván Gómez-Blázquez, Zakir H. Sahul, Ameer Kabour, Luisa Salido, Michael Cleman, Shigeru Saito, Martin B. Leon, Andreas Baumbach, PIONEER III Trial Investigators

**Affiliations:** a Institute of Cardiovascular Science, University College London, London, UK; b Barts Heart Centre, London, UK; c Division of Cardiology, Yale School of Medicine, New Haven, Connecticut; d Centre for Cardiovascular Medicine and Devices, William Harvey Research Institute, Queen Mary University of London, London, UK; e The Christ Hospital and the Lindner Research Center, Cincinnati, Ohio; f Department of Cardiology, Bern University Hospital, Inselspital, University of Bern, Bern, Switzerland; g Cardivascular Research Foundation, New York, New York; h University of Calgary, Calgary, Alberta, Canada; i Cardiology Associates of North Mississippi, Tupelo, Mississippi; j North Carolina Heart and Vascular, University of North Carolina, Raleigh, North Carolina; k Maasstad Ziekenhuis, Rotterdam, the Netherlands; l Hospital Álvaro Cunqueiro, Vigo, Spain; m AnMed Health Medical Center, Anderson, South Carolina; n NewYork-Presbyterian Hospital/Columbia University Medical Center, New York, New York; o Department of Cardiology, Imeldaziekenhuis, Bonheiden, Belgium; p Ziekenhuis Oost-Limburg, Genk, Belgium; q Hospital 12 de Octubre, Madrid, Spain; r Michigan Heart Ann Arbor, Ypsilanti, Michigan; s Mercy Health – St Vincent Medical Center, Toledo, Ohio; t University Hospital Ramon y Cajal de Madrid, Madrid, Spain; u Shonan Kamakura General Hospital, Kamakura, Japan

**Keywords:** Drug-eluting stent, Percutaneous coronary intervention, Coronary revascularization, Coronary artery disease, Acute coronary syndrome

## Abstract

**Background:**

The Supreme healing-targeted drug-eluting stent (DES) is designed to promote endothelial healing to reduce stent-related adverse events. This may be particularly relevant among complex lesions that have a higher rate of adverse events. We sought to compare 1-year outcomes of percutaneous coronary intervention in complex lesions between the Supreme DES and contemporary durable-polymer, everolimus-eluting stents (DP-EES).

**Methods:**

PIONEER III was a multicenter, prospective, single-blind clinical trial, randomizing 1629 patients with either an acute or chronic coronary syndrome in a 2:1 ratio to the Supreme DES or DP-EES. Complex lesions (American College of Cardiology/American Heart Association type B2/C) were found in 1137 patients. Outcomes were also compared for specific parameters of lesion complexity: severe calcification, long length (>20 ​mm), and severe tortuosity. The primary end point was target lesion failure at 1 ​year.

**Results:**

At 1 ​year, there was no difference in target lesion failure between the Supreme DES and DP-EES: (5.7% vs 5.6%; hazard ratio 1.00, 95% confidence interval 0.59-1.68, *P* = .99). Similarly, there were no differences in the secondary end points of lesion success (99.7% vs 99.4%, *P* = .41), device success (97.0% vs 98.5%, *P* = .14), target vessel failure (6.5% vs 7.4%, *P* = .50), major adverse cardiac events (7.8% vs 8.5%, *P* = .64), or stent thrombosis (0.7% vs 1.1%, *P* = .48). A trend was observed toward a higher rate of target lesion revascularization with the Supreme DES (2.5% vs 0.9%, *P* = .06).

**Conclusions:**

This study suggests that the Supreme DES is as effective and safe at 1 ​year compared with the standard DP-EES across a broad spectrum of lesion complexity.

## Central Illustration

**Figure:**
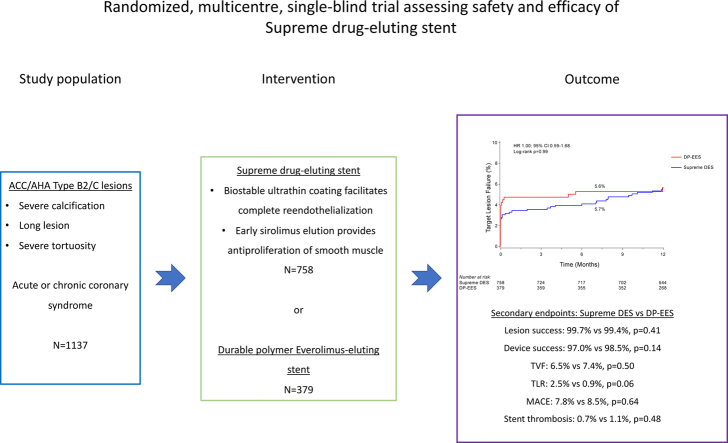

## Introduction

Advances in drug-eluting stent (DES) technology have reduced the incidence of adverse events associated with percutaneous coronary intervention (PCI), with notable reductions in stent thrombosis, in-stent restenosis, and myocardial infarction.^1^^,^^2^ A novel stent, the Supreme healing-targeted DES (SINOMED), uses technology to improve healing of the endothelium to restore vascular function and reduce adverse events.^3^ In a noninferiority randomized controlled trial (PIONEER III; NCT03168776), among patients with acute and chronic coronary syndromes (excluding ST-segment elevation myocardial infarction), PCI with the Supreme DES was noninferior to durable-polymer everolimus-eluting stents (DP-EES)^3^; however, a specific analysis of complex lesions has not been reported.

Lesions that are heavily calcified, long, or tortuous are common, technically challenging for PCI, and associated with higher rates of adverse events.^4^, ^5^, ^6^, ^7^ Mechanistically, adverse events after PCI are the result of inflammatory reactions, disruption of blood flow and endothelial function, and pathological remodeling involving vascular smooth muscle cell proliferation and fibrin deposition.^8^^,^^9^ Through quicker polymer degradation and drug release, which promote endothelial healing, the Supreme DES aims to restore vascular function, reduce inflammation, and curb stent-related adverse events.^10^ This substudy of PIONEER III compares outcomes between the Supreme DES and the DP-EES among patients with complex coronary lesions at 1 ​year after PCI.

## Methods

PIONEER III was a randomized, single-blind, multicenter, noninferiority trial comparing the safety and efficacy of the Supreme DES against the DP-EES (Xience or Promus DES). The trial design and inclusion and exclusion criteria can be found elsewhere.^3^ The trial was conducted in compliance with US Food and Drug Administration regulations, International Conference on Harmonization Good Clinical Practice guidelines, and the Declaration of Helsinki. All patients provided written informed consent. The protocol was approved by the applicable institutional review board or ethics committee at each center.

### Complex lesion analysis

All coronary angiograms were analyzed by an independent angiographic core laboratory (Yale Cardiovascular Research Group, Yale School of Medicine), blinded to treatment allocation, for baseline lesion characteristics and complexity. For this prespecified, but not stratified, substudy, complex lesions were defined as type B2 or type C lesions by the American College of Cardiology/American Heart Association guidelines.^6^^,^^11^ The specific lesion characteristics identified for this study included long lesions (≥20 ​mm), severe calcification, and excessive tortuosity of a proximal segment based on the independent angiographic core laboratory evaluation. Calcification was defined as readily apparent radiodensities noted within the vascular wall at the site of a stenosis was classified as severe when radiopacities were noted without cardiac motion before contrast injection and involved both sides of the arterial wall. Vessel tortuosity is defined as the number of bends that must be traversed by the balloon or device to reach the target lesion. Severe tortuosity is defined as at least 2 bends >90 degrees.

Any patient with at least one of these complex lesions treated with either the Supreme DES or DP-EES was included in this substudy.

### Outcomes

The primary end point was 12-month device-related target lesion failure (TLF), which is a composite of cardiac death, target vessel myocardial infarction, or clinically driven target lesion revascularization (TLR). The secondary end points included the following: (i) lesion success, defined as attainment of <30% residual stenosis of the target lesion (measured by quantitative coronary angiography) using any percutaneous method; (ii) device success, defined as lesion success using the assigned device; (iii) target vessel failure, defined as the composite of cardiac death, target vessel myocardial infarction, or target vessel revascularization; (iv) TLR; (v) major adverse cardiac events (MACE), defined as the composite of all-cause death, myocardial infarction as per the modified third universal definition,^12^ or target vessel revascularization; and (vi) stent thrombosis, defined by the Academic Research Consortium at all time points. The values reported here include the composite of definite, probable, and possible thrombosis.^13^ All protocol-defined end points were adjudicated by an independent clinical event committee (Cardiovascular Research Foundation).

### Statistical analysis

The intention-to-treat population was used for the primary and all secondary end point analyses. Categorical variables are reported as counts (percentages) and compared between treatment groups using χ^2^ or the Fisher exact test, as appropriate. Parametric continuous variables are presented as mean ​± ​standard deviation and compared with a 2-sample *t* test. Nonparametric data, determined using the Shapiro–Wilk test, were compared using the Wilcoxon rank-sum test and reported as median (first quartile, third quartile). Time-to-event outcomes were calculated using Kaplan–Meier methods and compared between groups using the log-rank test. Cox proportional hazards analysis was used to calculate hazard ratios with 95% confidence intervals and *P* values. A 2-sided *P* value <.05 was considered statistically significant without adjustment for multiplicity. Statistical analyses were carried out using SAS, version 9.4, (SAS Institute) by the Cardiovascular Research Foundation.

## Results

### Study population

Between October 2017 and July 2019, a total of 1629 patients were randomly assigned in a 2:1 ratio to the Supreme DES (1086 patients with 1304 lesions) or DP-EES (543 patients with 677 lesions). Of these, 1137 (70%) patients had complex lesions and were included; 161 (14.2%) patients had severely calcified lesions (104 with the Supreme DES and 57 with the DP-EES), 392 (34.5%) patients had long lesions (268 with the Supreme DES and 124 with the DP-EES), and 95 (8.4%) patients had severely tortuous lesions (60 with the Supreme DES and 35 with the DP-EES).

### Baseline characteristics

Baseline characteristics of the overall population and both study cohorts are shown in Table 1. The median age was 66 (58, 72) years, 75.4% were male, and the median body mass index was 28.3 (25.2, 32.0) kg/m^2^. Comorbidities and symptomatic status (New York Heart Association and Canadian Cardiovascular Society) were similar between both groups. Preprocedural indications were similar between both groups, with 51% presenting with stable angina, 19% with unstable angina, 9% with silent ischemia, and 21% with non–ST-segment elevation myocardial infarction.

**Table 1:** Baseline characteristics of patients with complex coronary lesions.

Characteristic	Overall (N ​= ​1137)	Supreme DES (n ​= ​758)	DP-EES (n ​= ​379)	*P* value
Age, years	66 (58, 72)	66 (58, 72)	65 (57, 72)	.65
Male sex	75.4% (857/1137)	76.9% (583/758)	72.3% (274/379)	.09
Body mass index, kg/m^2^	28.3 (25.2, 32.0)	28.3 (25.3, 31.7)	28.4 (25.2, 32.4)	.65
Comorbidities				
Diabetes mellitus	31.2% (355/1137)	32.3% (245/758)	29.0% (110/379)	.26
Type I	4.8% (17/355)	4.9% (12/245)	4.5% (5/110)	.89
Type II	95.2% (338/355)	95.1% (233/245)	95.5% (105/110)	.89
Hypertension	73.0% (830/1137)	74.3% (563/758)	70.4% (267/379)	.17
Hyperlipidemia	77.0% (876/1137)	76.9% (583/758)	77.3% (293/379)	.88
Renal disease	7.4% (84/1137)	7.5% (57/758)	7.1% (27/379)	.81
Family history of premature CAD^a^	35.6% (405/1137)	35.1% (266/758)	36.7% (139/379)	.60
Prior myocardial infarction	19.1% (217/1137)	19.1% (145/758)	19.0% (72/379)	.96
Prior PCI	30.1% (342/1137)	29.9% (227/758)	30.3% (115/379)	.89
Prior CABG	5.4% (61/1137)	5.4% (41/758)	5.3% (20/379)	.93
Prior stroke	4.2% (48/1137)	4.5% (34/758)	3.7% (14/379)	.53
Heart failure	4.6% (52/1137)	4.6% (35/758)	4.5% (17/379)	.92
Preserved ejection fraction (≥50%)	59.6% (28/47)	61.3% (19/31)	56.3% (9/16)	.74
Reduced ejection fraction (<50%)	40.4% (19/47)	38.7% (12/31)	43.8% (7/16)	.74
Atrial fibrillation	2.5% (28/1137)	2.0% (15/758)	3.4% (13/379)	.14
Smoking status				
Current	21.3% (242/1137)	21.0% (159/758)	21.9% (83/379)	.72
Former	38.0% (432/1137)	39.7% (301/758)	34.6% (131/379)	.09
NYHA class				
I	39.2% (20/51)	42.9% (15/35)	31.3% (5/16)	.43
II	35.3% (18/51)	34.3% (12/35)	37.5% (6/16)	.82
III	25.5% (13/51)	22.9% (8/35)	31.3% (5/16)	.73
IV	0.0% (0/51)	0.0% (0/35)	0.0% (0/16)	—
Chronic angina pectoris (before index event)	21.7% (247/1137)	21.6% (164/758)	21.9% (83/379)	.92
CCS class				
I	15.8% (39/247)	12.8% (21/164)	21.7% (18/83)	.07
II	39.3% (97/247)	39.0% (64/164)	39.8% (33/83)	.91
III	38.1% (94/247)	40.2% (66/164)	33.7% (28/83)	.32
IV	6.9% (17/247)	7.9% (13/164)	4.8% (4/83)	.36
Echocardiographic findings				
Left ventricular ejection fraction, %	57.57 ​± ​8.80	57.56 ​± ​8.87	57.60 ​± ​8.65	.95
Pre-procedure indication				
Stable angina	50.9% (579/1137)	49.9% (378/758)	53.0% (201/379)	.31
Unstable angina	19.3% (220/1137)	19.3% (146/758)	19.5% (74/379)	.92
Silent ischemia	9.0% (102/1137)	9.8% (74/758)	7.4% (28/379)	.19
NSTEMI	20.8% (236/1137)	21.1% (160/758)	20.1% (76/379)	.68

Values are median (first quartile, third quartile), mean ​± ​standard deviation, or % (n/N).

CABG, coronary artery bypass grafting; CAD, coronary artery disease; CCS, Canadian Cardiovascular Society; NSTEMI, non–ST-segment elevation myocardial infarction; NYHA, New York Heart Association; PCI, percutaneous coronary intervention.

a First-degree relative.

### Procedural characteristics

Target vessel treatment was similar between both groups except right coronary artery involvement was less common in the Supreme DES cohort compared with the DP-EES cohort (30.6% vs 36.7%, *P* = .019) (Table 2). Only 1 patient in this study (within the Supreme DES cohort) had a target lesion in a protected left main stem. Additional use of a device before balloon angioplasty was rare (0.6% in both cohorts), and the median number of stents implanted was 1.0 (1.0, 1.0)—the same in both cohorts. There was a high rate of radial access used in both cohorts (81.7% in the Supreme DES and 77.0% in the DP-EES).

**Table 2:** Procedural details of patients with complex coronary lesions.

Characteristic	Overall (N ​= ​1137 patients, 1447 lesions)	Supreme DES (n ​= ​758 patients, 954 lesions)	DP-EES (n ​= ​379 patients, 493 lesions)	*P* value
Vascular access site				
Femoral	19.3% (219/1137)	17.8% (135/758)	22.2% (84/379)	.08
Radial	80.1% (911/1137)	81.7% (619/758)	77.0% (292/379)	.07
Brachial	0.6% (7/1137)	0.5% (4/758)	0.8% (3/379)	.69
Baseline vessel characteristics				
Target vessel				
Left anterior descending	41.5% (601/1447)	42.8% (408/954)	39.1% (193/493)	.19
Left circumflex	25.7% (372/1447)	26.5% (253/954)	24.1% (119/493)	.33
Right	32.7% (473/1447)	30.6% (292/954)	36.7% (181/493)	.019
Protected left main	0.1% (1/1447)	0.1% (1/954)	0.0% (0/493)	1.00
Diameter stenosis, %	85.0 (80.0, 90.0)	85.0 (80.0, 90.0)	85.0 (80.0, 90.0)	.73
Baseline TIMI flow				
0	0.1% (1/1445)	0.1% (1/953)	0.0% (0/492)	1.00
1	3.5% (50/1445)	3.9% (37/953)	2.6% (13/492)	.22
2	8.4% (121/1445)	7.9% (75/953)	9.3% (46/492)	.34
3	88.1% (1273/1445)	88.1% (840/953)	88.0% (433/492)	.94
2 or 3	96.5% (1394/1445)	96.0% (915/953)	97.4% (479/492)	.19
Intravascular diagnostic utility				
Fractional flow reserve performed	7.7% (112/1447)	7.2% (69/954)	8.7% (43/493)	.31
Fractional flow reserve ≤0.8	73.2% (82/112)	76.8% (53/69)	67.4% (29/43)	.28
Intravascular ultrasound used	15.3% (221/1447)	14.7% (140/954)	16.4% (81/493)	.38
Lesion pretreatment				
Predilatation of the target vessel	76.8% (1111/1447)	79.4% (757/954)	71.8% (354/493)	.001
Pretreatment with a device other than balloon angioplasty	0.6% (9/1446)	0.6% (6/953)	0.6% (3/493)	1.00
Atherectomy	11.1% (1/9)	16.7% (1/6)	0.0% (0/3)	1.00
Cutting/scoring balloon	55.6% (5/9)	33.3% (2/6)	100.0% (3/3)	.17
Laser	11.1% (1/9)	16.7% (1/6)	0.0% (0/3)	1.00
Thrombectomy/aspiration	22.2% (2/9)	33.3% (2/6)	0.0% (0/3)	.50
Treatment characteristics				
Procedure duration, minutes	43.0 (30.0, 60.0)	44.0 (30.0, 62.0)	42.0 (28.0, 60.0)	.11
Number of stents implanted per target lesion	1.0 (1.0, 1.0)	1.0 (1.0, 1.0)	1.0 (1.0, 1.0)	.25
Total stent length, mm	20.0 (15.0, 30.0)	20.0 (15.0, 30.0)	23.0 (15.0, 28.0)	.32
Stent postdilated	52.2% (749/1436)	51.6% (489/947)	53.2% (260/489)	.58
Number of target lesions treated	1 (1, 1)	1 (1, 1)	1 (1, 2)	.22
Number of study stents implanted per subject	1 (1, 2)	1.0 (1, 2)	1 (1, 2)	.07
Total stent length, mm	28 (20, 35)	25 (20, 35)	28 (18, 38)	.65

Values are % (n/N) or median (first quartile, third quartile).

DES, drug-eluting stent; DP-EES, durable-polymer everolimus-eluting stent; TIMI, thrombolysis in myocardial infarction.

### Outcomes for PCI in patients with complex vs noncomplex lesions

At 1 ​year, outcomes with PCI were similar in complex compared with noncomplex lesions. There was a nonsignificant trend toward higher cardiovascular (0.7 vs 0.0%; *P* = .06) and cardiac mortality (0.6 vs 0.0%; *P* = .08) among the complex lesion cohort (Supplemental Table S1). Further analysis based on lesion subtype demonstrated similar outcomes with PCI between long lesions compared with short lesions and severely tortuous lesions compared with mild/moderately tortuous lesions (Supplemental Tables S2 and S3).

PCI in heavily calcified lesions compared with mild/moderately calcified lesions resulted in a trend toward higher TLF (8.1% vs 4.9%, *P* = .07), higher MACE (12.5% vs 6.8%, *P* = .007), target vessel revascularization (6.3% vs 3.2%; *P* = .04), and bleeding rates (5.7% vs 2.7%; *P* = .034) and lower device success (93.3% vs 98.3%, *P* = .0005) (Supplemental Table S4).

### Primary outcome among all patients with complex lesions

**Fig. 1:**
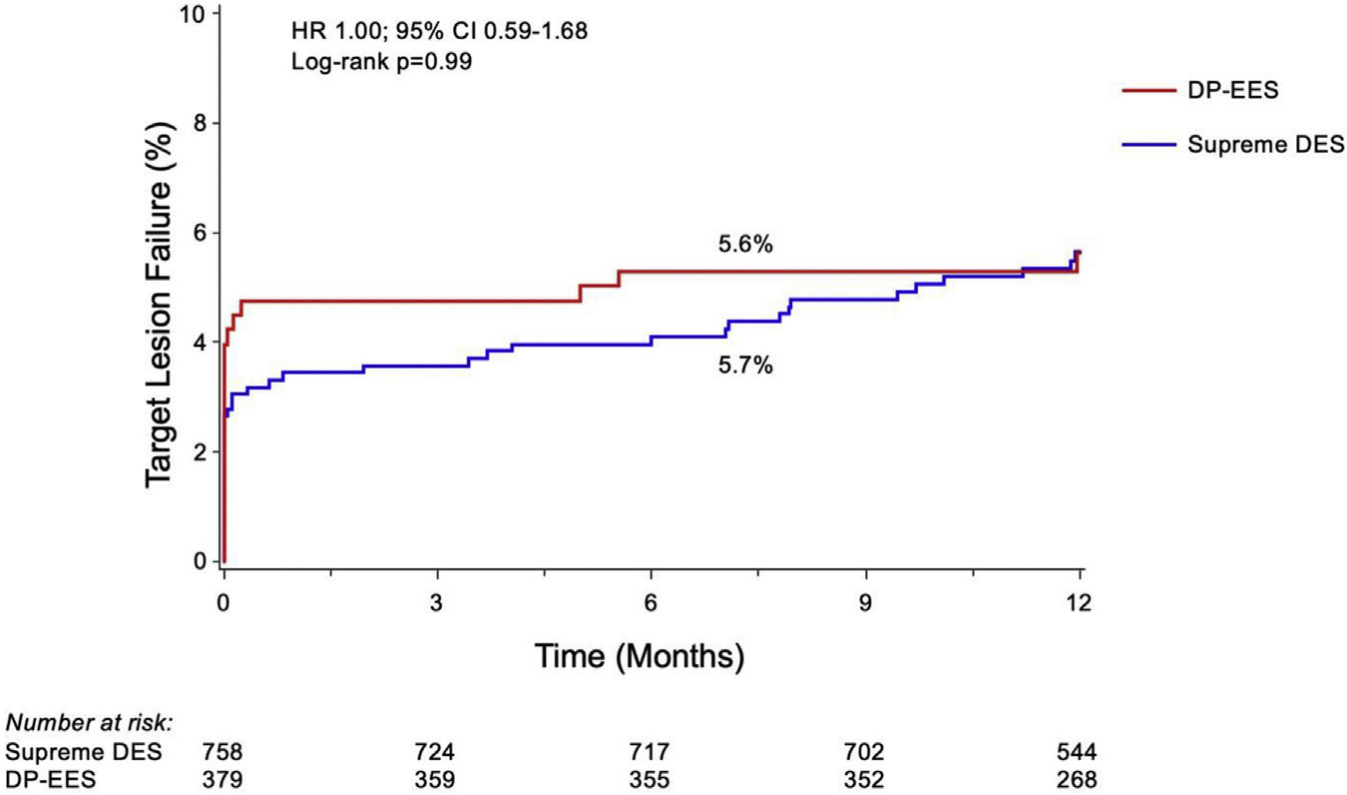
Target Lesion Failure Between the Supreme Drug-Eluting Stent and the Durable-Polymer Everolimus-Eluting Stent Among Patients With Complex Coronary Lesions. CI, confidence interval; DES, drug-eluting stent; DP, durable polymer; EES, everolimus-eluting stent; HR, hazard ratio.

There was no significant difference in the primary end point of TLF at 12 ​months between the Supreme DES and the DP-EES (5.7% vs 5.6%; hazard ratio 1.00, 95% confidence interval 0.59-1.68, *P* = .99) (Fig. 1). The individual components of the primary end point were similar between the Supreme DES and DP-EES cohorts including cardiac death (0.4 vs 1.1%; *P* = .18) and target vessel myocardial infarction (3.3 vs 4.5%; *P* = .32), whereas a trend was observed toward a higher rate of clinically driven TLR (2.3 vs 0.9%; *P* = .08) (Table 3).

**Central Illustration:**
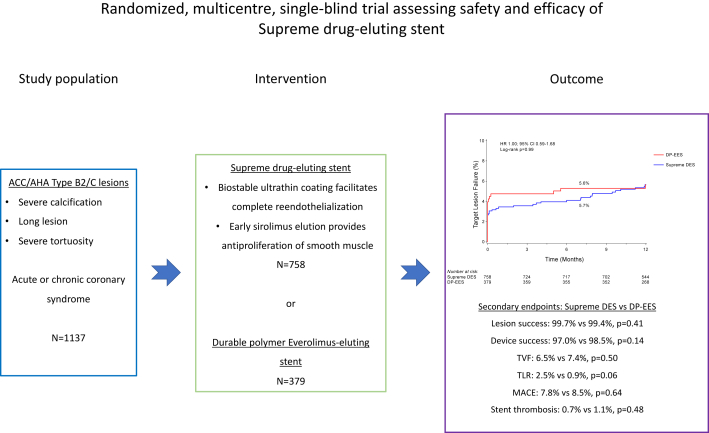
Summary of Trial Findings. ACC, American College of Cardiology; AHA, American Heart Association; CI, confidence interval; DES, drug-eluting stent; DP-EES, durable polymer everolimus-eluting stent; HR, hazard ratio.

**Table 3:** Comparison of outcomes between the supreme DES and the DP-EES among patients with complex coronary lesions.

Outcome	Overall (N ​= ​1137)	Supreme DES (n ​= ​758)	DP-EES (n ​= ​379)	Hazard ratio [95% confidence interval]	*P* value
Primary end point					
Target lesion failure	5.6% (63)	5.7% (42)	5.6% (21)	1.00 [0.59, 1.68]	.99
Secondary end points					
Lesion success	99.6% (1401/1407)	99.7% (927/930)	99.4% (474/477)		.41
Device success	97.5% (1344/1378)	97.0% (879/906)	98.5% (465/472)		.14
Target vessel failure	6.8% (76)	6.5% (48)	7.4% (28)	0.85 [0.53, 1.36]	.50
Target lesion revascularization with PCI	2.0% (21)	2.5% (18)	0.9% (3)	3.03 [0.89, 10.30]	.06
Major adverse cardiac events	8.0% (90)	7.8% (58)	8.5% (32)	0.90 [0.59, 1.39]	.64
Stent thrombosis	0.8% (9)	0.7% (5)	1.1% (4)	0.63 [0.17, 2.33]	.48
Components of end points					
All-cause mortality	1.1% (12)	0.7% (5)	1.9% (7)	0.36 [0.11, 1.13]	.07
Cardiovascular death	0.7% (8)	0.5% (4)	1.1% (4)	0.50 [0.13, 2.00]	.32
Cardiac death	0.6% (7)	0.4% (3)	1.1% (4)	0.38 [0.08, 1.68]	.18
Myocardial infarction	5.0% (56)	4.9% (37)	5.0% (19)	0.97 [0.56, 1.69]	.91
Periprocedural	3.3% (38)	2.8% (21)	4.5% (17)	0.62 [0.33, 1.17]	.13
Nonperiprocedural	1.9% (21)	2.3% (17)	1.1% (4)	2.14 [0.72, 6.36]	.16
Target vessel myocardial infarction	3.7% (42)	3.3% (25)	4.5% (17)	0.73 [0.40, 1.36]	.32
Periprocedural	3.1% (35)	2.5% (19)	4.2% (16)	0.59 [0.31, 1.15]	.11
Nonperiprocedural	0.9% (10)	1.0% (7)	0.8% (3)	1.17 [0.30, 4.52]	.82
Any revascularization	5.0% (55)	5.2% (38)	4.6% (17)	1.12 [0.63, 1.99]	.69
PCI	4.8% (53)	5.0% (37)	4.4% (16)	1.16 [0.65, 2.09]	.61
CABG	0.3% (3)	0.3% (2)	0.3% (1)	1.00 [0.09, 11.05]	1.00
Target vessel revascularization	3.6% (40)	3.7% (27)	3.5% (13)	1.04 [0.54, 2.02]	.91
PCI	3.5% (39)	3.5% (26)	3.5% (13)	1.00 [0.51, 1.95]	1.00
CABG	0.1% (1)	0.1% (1)	0.0% (0)	—	.48
Clinically driven target lesion revascularization	1.9% (20)	2.3% (17)	0.9% (3)	2.86 [0.84, 9.77]	.08
Early stent thrombosis (0 to 30 ​days after stent implantation)	0.6% (7)	0.7% (5)	0.5% (2)	1.25 [0.24, 6.45]	.79
Acute stent thrombosis (≤1 ​day)	0.2% (2)	0.1% (1)	0.3% (1)	0.50 [0.03, 7.99]	.62
Subacute stent thrombosis (2-30 ​days)	0.4% (5)	0.5% (4)	0.3% (1)	2.01 [0.22, 17.94]	.53
Late stent thrombosis (>30 ​days to 1 ​year after stent implantation)	0.2% (2)	0.0% (0)	0.5% (2)	—	.046
Other complications					
Bleeding (BARC definition)	3.3% (37)	3.1% (23)	3.7% (14)	0.82 [0.42, 1.59]	.55
Type 3 or type 5	1.9% (21)	2.3% (17)	1.1% (4)	2.14 [0.72, 6.35]	.16

Values are % (n) or % (n/N). The hazard ratios are Kaplan-Meier estimates based on the time to event for each cohort.

BARC, Bleeding Academic Research Consortium; CABG, coronary artery bypass grafting; DES, drug-eluting stent; DP-EES, durable-polymer everolimus-eluting stent; PCI, percutaneous coronary intervention.

### Secondary outcomes among all patients with complex lesions

All secondary outcomes were similar between the Supreme DES and DP-EES including lesion success (99.7 vs 99.4%, *P* = .41) and device success (97.0 vs 98.5%, *P* = .14). There were no differences in target vessel failure (6.5% vs 7.4%, *P* = .50), TVR (3.7% vs 3.5%, *P* = .91), MACE (7.8% vs 8.5%, *P* = .64), or stent thrombosis (0.7% vs 1.1%, *P* = .48) between the Supreme DES and the DP-EES, and no reinterventions required surgery. A nonsignificant trend was observed for higher TLR with the Supreme DES vs DP-EES (2.5 vs 0.9%, *P* = .06). All-cause mortality at 1 ​year with the Supreme DES compared with the DP-EES was 0.7% vs 1.9%, *P* = .07. Late stent thrombosis with the Supreme DES was 0.0% vs 0.5% with the DP-EES, *P* = .046. All other outcomes are reported in Table 3.

### Secondary outcomes based on specific lesion complexity criteria

For patients with long lesions, TLF was similar between the Supreme DES and the DP-EES (5.5% vs 5.7%, *P* = .86), and all other end points were similar between the 2 groups (Supplemental Table S5). Among severely tortuous lesions, TLF was similar between the Supreme DES and the DP-EES (6.8% vs 8.6%, *P* = .72). All other end points were similar between the 2 groups (Supplemental Table S6).

For patients with severely calcified lesions, TLF was similar between the Supreme DES and the DP-EES (8.8% vs 7.0%, *P* = .72), with a nonsignificant trend for higher TLR with the Supreme DES (4.9% vs 0.0%, *P* = .09) (Supplemental Table S7). The remaining secondary end points were comparable between the 2 cohorts. Overall bleeding rates with the Supreme DES compared with the DP-EES cohort were 2.9% vs 10.6%, respectively, *P* = .045.

## Discussion

This multicenter, randomized controlled trial compared outcomes in patients with complex coronary lesions treated with a Supreme DES or DP-EES. This study demonstrated two important findings. First, TLF at 1 ​year after PCI was similar between the Supreme DES and the DP-EES. Second, PCI in heavily calcified lesions, but not long lesions or severely tortuous lesions, was associated with more adverse outcomes.

Most end points were comparable between the Supreme DES and the DP-EES (Central Illustration); however, some differences are worth noting. All-cause mortality trended toward lower rates with the Supreme DES, with comparable rates of cardiovascular and cardiac death between both cohorts. However, this study was not powered to assess this end point, and therefore, inferences cannot be made from these data. Late stent thrombosis was lower with the Supreme DES; however, with a low number of such events (n = 2), more early stent thrombosis with the Supreme DES, and no difference in overall stent thrombosis, inferences about late stent thrombosis cannot be made. Among patients with severe calcification, bleeding rates were lower with the Supreme DES but similar among other complex coronary lesions. This requires further evaluation of antiplatelet regimes and bleeding risk between the cohorts. From an efficacy perspective, a trend was observed toward higher TLR with the Supreme DES compared with the DP-EES. This numerically higher rate was seen with all 3 lesion types; however, this finding should be interpreted with caution. The rate of TLR for the DP-EES reported in other studies (1.8-2.4%) is higher than that in our study.^14^^,^^15^

The genesis of the Supreme DES stems from the need to curb stent-related clinical events, which are common. Within the first year after PCI, registry data (with half the study population exhibiting American College of Cardiology/American Heart Association type C lesions) demonstrated that 8% of patients require repeat PCI, of which approximately half do so in the target vessel.^16^ Among pathological factors, neoatherosclerosis, inflammation, and delayed vessel healing account for a TLF rate of 7.7% within 1-5 ​years of PCI for both complex and noncomplex lesions with the second-generation DES.^17^ Mechanistically, after delivery of the antiproliferative drug by the DP-EES, the exposed polymer promotes inflammation, which impairs arterial healing and stent endothelialization and contributes to neoatherosclerosis. These processes may lead to late stent thrombosis and restenosis.^10^ The biodegradable polymer in Supreme DES overcomes this by degrading faster than most polymers, enabling quicker healing of the vasculature and hence restoring functional endothelium and preventing associated inflammation, thereby reducing adverse events. Complex lesions are associated with higher rates of in-stent restenosis and stent thrombosis, commonly due to stent underexpansion,^18^, ^19^, ^20^ and account for most stent failures.^21^ The Supreme DES may be well suited for this higher-risk cohort. Our results support those of others,^7^^,^^22^ who showed that PCI in moderate-to-severe calcified lesions is associated with adverse outcomes compared with PCI in mildly calcified lesions regardless of the generation of the stent. However, contrary to previous studies,^6^^,^^23^ event rates in our study did not differ between long and short lesions nor between severely tortuous lesions compared with mild/moderately tortuous lesions. It should be noted that fewer stents per patient were used in this study (median 1), than in the above mentioned studies, potentially suggesting a lower burden of complex lesions in our study.

Initial studies suggested higher late lumen loss with the Supreme DES than with other DESs^24^^,^^25^; however, this did not translate into restenosis or impaired functional results when measured by quantitative flow ratio.^26^ The recently published PIONEER III study established the stent's safety and efficacy,^3^ whereas the present study lends support to the stent's use in a high-risk population; however, long-term results are awaited to provide further evidence of improved late outcomes and proof of concept of a healing-targeted stent. In addition to the safety and efficacy of the Supreme DES, costing and availability will play an important role in light of the tender and national approval processes. The cost of the Supreme DES will be approximately $280.00, and commercial use of the Supreme DES has started in Ireland with availability set to expand.^27^

### Limitations

The study was powered for non-inferiority of TLF in the overall population and was not powered for any of the secondary end points. In addition, although the substudy presented here was prespecified, patients were not stratified as per lesion complexity. The complexity of coronary artery disease in our population was relatively moderate and so our results may not be applicable to patients with severely complex coronary artery disease. The premise of the Supreme DES to promote endothelial healing may only unfold during longer-term follow-up; therefore, 12-month outcomes may not demonstrate the full potential of the Supreme DES, and long-term outcomes are awaited. Finally, the study excluded patients with ST-segment elevation MI, left main stem disease, and chronic total occlusions, limiting its generalizability.

## Conclusions

This substudy of the PIONEER III study suggests that the Supreme DES is as safe and effective as the DP-EES at 1 ​year for the treatment of complex coronary lesions.
