# Supplementary material for: Outcomes of the Novel Supreme Drug-Eluting Stent in Complex Coronary Lesions: A PIONEER III Substudy
Source: J Soc Cardiovasc Angiogr Interv. 2022 Jan 30;1(1):100004. doi: 10.1016/j.jscai.2021.100004 (PMC11308031; doi:10.1016/j.jscai.2021.100004)
Supplement: Supplemental Tables S1-S7 [file mmc1.docx]

**Supplementary tables**

Supplementary table 1: Outcomes with PCI in complex vs non-complex lesions

| **Parameter** | **Complex Lesions (N=1137)** | **Non-Complex Lesions (N=486)** | **Hazard Ratio** | **P-value** |
| --- | --- | --- | --- | --- |
| **Primary endpoint** | | | | |
| **TLF** | 5.6% (63) | 4.3% (21) | 1.29 [0.79, 2.11] | 0.31 |
| **Secondary endpoints** | | | | |
| **Lesion Success** | 99.6% (1401/ 1407) | 99.8% (523/ 524) |  | 0.46 |
| **Device Success** | 97.5% (1344/ 1378) | 98.4% (508/ 516) |  | 0.26 |
| **TVF** | 6.8% (76) | 5.0% (24) | 1.37 [0.86, 2.17] | 0.18 |
| **Target Lesion Revascularization (TLR)** | 2.0% (21) | 2.1% (10) | 0.89 [0.42, 1.89] | 0.77 |
| **MACE** | 8.0% (90) | 5.8% (28) | 1.40 [0.91, 2.13] | 0.12 |
| **Stent Thrombosis** | 0.8% (9) | 0.6% (3) | 1.29 [0.35, 4.75] | 0.70 |
| **Components of endpoints** | | | | |
| **All-Cause Mortality** | 1.1% (12) | 0.4% (2) | 2.57 [0.58, 11.50] | 0.20 |
| Cardiovascular Death | 0.7% (8) | 0.0% (0) | N/A | 0.06 |
| Cardiac Death | 0.6% (7) | 0.0% (0) | N/A | 0.08 |
| Non-Cardiovascular Death | 0.4% (4) | 0.4% (2) | 0.86 [0.16, 4.71] | 0.87 |
| **MI** | 5.0% (56) | 3.9% (19) | 1.27 [0.75, 2.14] | 0.36 |
| Peri-procedural MI | 3.3% (38) | 1.9% (9) | 1.81 [0.87, 3.74] | 0.10 |
| Non Peri-procedural MI | 1.9% (21) | 2.1% (10) | 0.90 [0.42, 1.91] | 0.78 |
| **Target Vessel MI** | 3.7% (42) | 3.3% (16) | 1.13 [0.63, 2.00] | 0.68 |
| Peri-procedural MI | 3.1% (35) | 1.9% (9) | 1.66 [0.80, 3.46] | 0.16 |
| Non Peri-procedural MI | 0.9% (10) | 1.4% (7) | 0.61 [0.23, 1.60] | 0.31 |
| **Any Revascularization** | 5.0% (55) | 4.2% (20) | 1.19 [0.71, 1.98] | 0.51 |
| PCI | 4.8% (53) | 3.7% (18) | 1.27 [0.74, 2.17] | 0.38 |
| CABG | 0.3% (3) | 0.4% (2) | 0.65 [0.11, 3.86] | 0.63 |
| **Target Vessel Revascularization (TVR)** | 3.6% (40) | 3.3% (16) | 1.07 [0.60, 1.92] | 0.81 |
| PCI | 3.5% (39) | 2.9% (14) | 1.20 [0.65, 2.21] | 0.56 |
| **TLR with PCI** | 2.0% (21) | 1.9% (9) | 0.99 [0.45, 2.17] | 0.98 |
| Clinically driven TLR | 1.9% (20) | 1.9% (9) | 0.94 [0.43, 2.07] | 0.89 |
| Acute Stent Thrombosis (<= 1day after stent implantation) | 0.2% (2) | 0.0% (0) | N/A | 0.36 |
| Subacute Stent Thrombosis (2-30 daysr after stent implantation) | 0.4% (5) | 0.4% (2) | 1.07 [0.21, 5.51] | 0.94 |
| Early Stent Thrombosis (0 to 30 days after stent implantation) | 0.6% (7) | 0.4% (2) | 1.50 [0.31, 7.21] | 0.61 |
| Late Stent Thrombosis (> 30 days to 1 year after stent implantation) | 0.2% (2) | 0.2% (1) | 0.86 [0.08, 9.49] | 0.90 |
| **Other complications** | | | | |
| **Bleeding (BARC Definition)** | 3.3% (37) | 2.3% (11) | 1.45 [0.74, 2.85] | 0.27 |
| Type 3 or Type 5 | 1.9% (21) | 1.7% (8) | 1.13 [0.50, 2.54] | 0.77 |

BARC = Bleeding Academic Research Consortium; CABG = coronary artery bypass grafting; PCI = percutaneous coronary intervention.

Supplementary table 2: Outcomes with PCI in long vs short lesions

| **Parameter** | **Long Lesion >20mm (N=392)** | **Short Lesion <20mm (N=1231)** | **Hazard Ratio** | **P-value** |
| --- | --- | --- | --- | --- |
| **Primary endpoint** | | | | |
| **TLF** | 5.5% (21) | 5.2% (63) | 1.05 [0.64, 1.72] | 0.86 |
| **Secondary endpoints** | | | | |
| **Lesion Success** | 99.2% (517/ 521) | 99.8% (1407/ 1410) | 99.6% (1924/ 1931) | 0.09 |
| **Device Success** | 97.2% (491/ 505) | 98% (1361/ 1389) | 97.8% (1852/ 1894) | 0.45 |
| **TVF** | 6.8% (26) | 6.1% (74) | 1.11 [0.71, 1.73] | 0.66 |
| **Target Lesion Revascularization (TLR)** | 1.9% (7) | 2.0% (24) | 0.91 [0.39, 2.11] | 0.83 |
| **MACE** | 9.1% (35) | 6.8% (83) | 1.34 [0.90, 1.99] | 0.14 |
| **Stent Thrombosis** | 0.8% (3) | 0.7% (9) | 1.05 [0.28, 3.88] | 0.94 |
| **Components of endpoints** | | | | |
| **All-Cause Mortality** | 1.0% (4) | 0.8% (10) | 1.26 [0.39, 4.01] | 0.70 |
| Cardiovascular Death | 0.8% (3) | 0.4% (5) | 1.88 [0.45, 7.88] | 0.38 |
| Cardiac Death | 0.5% (2) | 0.4% (5) | 1.25 [0.24, 6.47] | 0.79 |
| **MI** | 5.7% (22) | 4.3% (53) | 1.31 [0.80, 2.16] | 0.28 |
| Peri-procedural MI | 3.8% (15) | 2.6% (32) | 1.47 [0.80, 2.72] | 0.21 |
| Non Peri-procedural MI | 2.1% (8) | 1.9% (23) | 1.09 [0.49, 2.44] | 0.83 |
| **Target Vessel MI** | 3.6% (14) | 3.6% (44) | 1.00 [0.55, 1.82] | 1.00 |
| Peri-procedural MI | 3.1% (12) | 2.6% (32) | 1.18 [0.61, 2.29] | 0.62 |
| Non Peri-procedural MI | 0.8% (3) | 1.2% (14) | 0.67 [0.19, 2.34] | 0.53 |
| **Any Revascularization** | 4.8% (18) | 4.7% (57) | 0.99 [0.58, 1.68] | 0.97 |
| PCI | 4.8% (18) | 4.4% (53) | 1.07 [0.62, 1.82] | 0.82 |
| CABG | 0.0% (0) | 0.4% (5) | N/A | 0.21 |
| **Target Vessel Revascularization (TVR)** | 3.5% (13) | 3.6% (43) | 0.95 [0.51, 1.76] | 0.87 |
| PCI | 3.5% (13) | 3.3% (40) | 1.02 [0.55, 1.91] | 0.95 |
| **TLR with PCI** | 1.9% (7) | 1.9% (23) | 0.95 [0.41, 2.22] | 0.91 |
| Clinically driven TLR | 1.9% (7) | 1.8% (22) | 0.99 [0.42, 2.33] | 0.99 |
| Acute Stent Thrombosis (<= 1day after stent implantation) | 0.3% (1) | 0.1% (1) | 3.14 [0.20, 50.21] | 0.39 |
| Subacute Stent Thrombosis (2-30 days after stent implantation) | 0.3% (1) | 0.5% (6) | 0.52 [0.06, 4.36] | 0.54 |
| Early Stent Thrombosis (0 to 30 days after stent implantation) | 0.5% (2) | 0.6% (7) | 0.90 [0.19, 4.33] | 0.90 |
| Late Stent Thrombosis (> 30 days to 1 year after stent implantation) | 0.3% (1) | 0.2% (2) | 1.57 [0.14, 17.36] | 0.71 |
| **Other complications** | | | | |
| **Bleeding (BARC Definition)** | 3.3% (13) | 2.9% (35) | 1.17 [0.62, 2.21] | 0.62 |
| Type 3 or Type 5 | 1.5% (6) | 1.9% (23) | 0.82 [0.33, 2.00] | 0.66 |

BARC = Bleeding Academic Research Consortium; CABG = coronary artery bypass grafting; PCI = percutaneous coronary intervention.

Supplementary table 3: Outcomes with PCI in severe vs mild/moderate tortuous lesions

| **Parameter** | **Severe Tortuosity (N=95)** | **Mild/Moderate Tortuosity (N=1528)** | **Hazard Ratio** | **P-value** |
| --- | --- | --- | --- | --- |
| **Primary endpoint** | | | | |
| **TLF** | 7.4% (7) | 5.1% (77) | 1.47 [0.68, 3.18] | 0.33 |
| **Secondary endpoint** | | | | |
| **Lesion Success** | 100% (125/ 125) | 99.6% (1799/ 1806) |  | 0.98 |
| **Device Success** | 95.8% (115/ 120) | 97.9% (1737/ 1774) |  | 0.16 |
| **TVF** | 7.4% (7) | 6.2% (93) | 1.21 [0.56, 2.61] | 0.62 |
| **Target Lesion Revascularization (TLR)** | 1.1% (1) | 2.1% (30) | 0.53 [0.07, 3.90] | 0.53 |
| **MACE** | 7.4% (7) | 7.3% (111) | 1.01 [0.47, 2.17] | 0.97 |
| **Stent Thrombosis** | 2.1% (2) | 0.7% (10) | 3.23 [0.71, 14.73] | 0.11 |
| **Components of endpoints** | | | | |
| **All-Cause Mortality** | 1.1% (1) | 0.9% (13) | 1.23 [0.16, 9.41] | 0.84 |
| Cardiovascular Death | 1.1% (1) | 0.5% (7) | 2.29 [0.28, 18.62] | 0.43 |
| Cardiac Death | 1.1% (1) | 0.4% (6) | 2.67 [0.32, 22.16] | 0.34 |
| **MI** | 6.4% (6) | 4.6% (69) | 1.40 [0.61, 3.23] | 0.42 |
| Peri-procedural MI | 3.2% (3) | 2.9% (44) | 1.10 [0.34, 3.53] | 0.88 |
| Non Peri-procedural MI | 3.2% (3) | 1.9% (28) | 1.72 [0.52, 5.66] | 0.37 |
| **Target Vessel MI** | 5.3% (5) | 3.5% (53) | 1.52 [0.61, 3.81] | 0.36 |
| Peri-procedural MI | 3.2% (3) | 2.7% (41) | 1.18 [0.36, 3.80] | 0.78 |
| Non Peri-procedural MI | 2.1% (2) | 1.0% (15) | 2.15 [0.49, 9.38] | 0.30 |
| **Any Revascularization** | 3.2% (3) | 4.8% (72) | 0.67 [0.21, 2.12] | 0.49 |
| PCI | 3.2% (3) | 4.6% (68) | 0.71 [0.22, 2.25] | 0.56 |
| **Target Vessel Revascularization (TVR)** | 3.2% (3) | 3.6% (53) | 0.91 [0.29, 2.93] | 0.88 |
| PCI | 3.2% (3) | 3.4% (50) | 0.97 [0.30, 3.11] | 0.96 |
| **TLR with PCI** | 1.1% (1) | 2.0% (29) | 0.55 [0.07, 4.04] | 0.55 |
| Clinically driven TLR | 1.1% (1) | 1.9% (28) | 0.57 [0.08, 4.18] | 0.57 |
| Acute Stent Thrombosis (<= 1day after stent implantation) | 0.0% (0) | 0.1% (2) | N/A | 0.72 |
| Subacute Stent Thrombosis (2-30 days after stent implantation) | 2.1% (2) | 0.3% (5) | 6.48 [1.26, 33.41] | 0.010 |
| Early Stent Thrombosis (0 to 30 days after stent implantation) | 2.1% (2) | 0.5% (7) | 4.62 [0.96, 22.22] | 0.036 |
| Late Stent Thrombosis (> 30 days to 1 year after stent implantation) | 0.0% (0) | 0.2% (3) | N/A | 0.66 |
| **Other complications** | | | | |
| **Bleeding (BARC Definition)** | 4.3% (4) | 2.9% (44) | 1.46 [0.52, 4.06] | 0.47 |
| Type 3 or Type 5 | 3.3% (3) | 1.7% (26) | 1.86 [0.56, 6.15] | 0.30 |

BARC = Bleeding Academic Research Consortium; CABG = coronary artery bypass grafting; PCI = percutaneous coronary intervention.

Supplementary table 4: Outcomes with PCI in severe vs mild/moderately calcified coronary lesions

| **Parameter** | **Heavy Calcification (N=161)** | **Mild-Moderate Calcification (N=1462)** | **Hazard Ratio** | **P-value** |
| --- | --- | --- | --- | --- |
| **Primary endpoint** | | | | |
| **TLF** | 8.1% (13) | 4.9% (71) | 1.70 [0.94, 3.07] | 0.07 |
| **Secondary endpoints** | | | | |
| **Lesion Success** | 99.5% (196/ 197) | 99.7% (1728/ 1734) |  | 0.72 |
| **Device Success** | 93.3% (181/ 194) | 98.3% (1671/ 1700) |  | 0.0005 |
| **TVF** | 11.3% (18) | 5.7% (82) | 2.06 [1.24, 3.43] | 0.004 |
| **Target Lesion Revascularization (TLR)** | 3.2% (5) | 1.9% (26) | 1.79 [0.69, 4.66] | 0.23 |
| **MACE** | 12.5% (20) | 6.8% (98) | 1.92 [1.19, 3.10] | 0.007 |
| **Stent Thrombosis** | 1.2% (2) | 0.7% (10) | 1.84 [0.40, 8.42] | 0.42 |
| **Components of endpoints** | | | | |
| **All-Cause Mortality** | 1.9% (3) | 0.8% (11) | 2.51 [0.70, 8.98] | 0.14 |
| Cardiovascular Death | 0.6% (1) | 0.5% (7) | 1.31 [0.16, 10.66] | 0.80 |
| Cardiac Death | 0.6% (1) | 0.4% (6) | 1.53 [0.18, 12.71] | 0.69 |
| Non-Cardiovascular Death | 1.3% (2) | 0.3% (4) | 4.59 [0.84, 25.08] | 0.053 |
| **MI** | 6.2% (10) | 4.5% (65) | 1.42 [0.73, 2.76] | 0.30 |
| Peri-procedural MI | 5.0% (8) | 2.7% (39) | 1.87 [0.87, 4.00] | 0.10 |
| Non Peri-procedural MI | 1.9% (3) | 2.0% (28) | 0.99 [0.30, 3.25] | 0.99 |
| **Target Vessel MI** | 5.6% (9) | 3.4% (49) | 1.68 [0.83, 3.43] | 0.14 |
| Peri-procedural MI | 5.0% (8) | 2.5% (36) | 2.02 [0.94, 4.36] | 0.06 |
| Non Peri-procedural MI | 1.2% (2) | 1.1% (15) | 1.23 [0.28, 5.37] | 0.78 |
| **Any Revascularization** | 7.6% (12) | 4.4% (63) | 1.79 [0.96, 3.31] | 0.06 |
| PCI | 7.0% (11) | 4.2% (60) | 1.71 [0.90, 3.26] | 0.10 |
| CABG | 0.6% (1) | 0.3% (4) | 2.29 [0.26, 20.50] | 0.45 |
| **Target Vessel Revascularization (TVR)** | 6.3% (10) | 3.2% (46) | 2.04 [1.03, 4.04] | 0.037 |
| PCI | 6.3% (10) | 3.0% (43) | 2.18 [1.10, 4.34] | 0.023 |
| **TLR with PCI** | 3.2% (5) | 1.8% (25) | 1.86 [0.71, 4.86] | 0.20 |
| Clinically driven TLR | 3.2% (5) | 1.7% (24) | 1.94 [0.74, 5.09] | 0.17 |
| Acute Stent Thrombosis (<= 1day after stent implantation) | 0.6% (1) | 0.1% (1) | 9.08 [0.57, 145.18] | 0.058 |
| Subacute Stent Thrombosis (2-30 days after stent implantation) | 0.6% (1) | 0.4% (6) | 1.53 [0.18, 12.70] | 0.69 |
| Early Stent Thrombosis (0 to 30 days after stent implantation) | 1.2% (2) | 0.5% (7) | 2.63 [0.55, 12.65] | 0.21 |
| Late Stent Thrombosis (> 30 days to 1 year after stent implantation) | 0.0% (0) | 0.2% (3) | N/A | 0.57 |
| **Other complications** | | | | |
| **Bleeding (BARC Definition)** | 5.7% (9) | 2.7% (39) | 2.15 [1.04, 4.44] | 0.034 |
| Type 3 or Type 5 | 1.3% (2) | 1.9% (27) | 0.68 [0.16, 2.84] | 0.59 |

BARC = Bleeding Academic Research Consortium; CABG = coronary artery bypass grafting; PCI = percutaneous coronary intervention.

**Supplemental Table 5. Comparison of Outcomes Between Supreme DES and DP-EES Among Patients With Long Lesions (>20 mm)**

| **Parameter** | **Supreme DES (n=268)** | **DP-EES (n=124)** | **Hazard Ratio [95% Confidence Interval]** | **p Value** |
| --- | --- | --- | --- | --- |
| **Primary endpoint** | | | | |
| Target lesion failure | 5.5% (14) | 5.7% (7) | 0.92 [0.37, 2.29] | 0.86 |
| **Secondary endpoints** | | | | |
| Lesion success | 99.4% (354/356) | 98.8% (163/165) |  | 0.44 |
| Device success | 95.9% (330/344) | 100% (161/161) |  | 0.98 |
| Target vessel failure | 6.6% (17) | 7.3% (9) | 0.87 [0.39, 1.95] | 0.74 |
| Target lesion revascularization with PCI | 2.5% (6) | 0.8% (1) | 2.82 [0.34, 23.45] | 0.31 |
| Major adverse cardiac events | 9.2% (24) | 8.9% (11) | 1.01 [0.50, 2.07] | 0.97 |
| Stent thrombosis | 0.4% (1) | 1.6% (2) | 0.23 [0.02, 2.55] | 0.19 |
| **Components of endpoints** | | | | |
| All-cause mortality | 0.8% (2) | 1.6% (2) | 0.47 [0.07, 3.32] | 0.44 |
| Cardiovascular death | 0.8% (2) | 0.8% (1) | 0.93 [0.08, 10.29] | 0.96 |
| Myocardial infarction | 5.7% (15) | 5.6% (7) | 0.99 [0.40, 2.43] | 0.98 |
| Periprocedural | 3.0% (8) | 5.7% (7) | 0.53 [0.19, 1.46] | 0.20 |
| Non-Periprocedural | 2.7% (7) | 0.8% (1) | 3.28 [0.40, 26.62] | 0.24 |
| Target vessel myocardial infarction | 3.1% (8) | 4.8% (6) | 0.62 [0.21, 1.77] | 0.36 |
| Revascularization with PCI | 5.1% (13) | 4.2% (5) | 1.22 [0.44, 3.43] | 0.70 |
| Target vessel revascularization with PCI | 3.6% (9) | 3.2% (4) | 1.05 [0.32, 3.41] | 0.94 |
| Early stent thrombosis (0-30 days after stent implantation) | 0.4% (1) | 0.8% (1) | 0.46 [0.03, 7.38] | 0.58 |
| Acute stent thrombosis (≤1 day after stent implantation) | 0.0% (0) | 0.8% (1) | — | 0.14 |
| Subacute stent thrombosis (2-30 days after stent implantation) | 0.4% (1) | 0.0% (0) | — | 0.50 |
| Late stent thrombosis (>30 days to 1 year after stent implantation) | 0.0% (0) | 0.8% (1) | — | 0.14 |
| **Other complications** | | | | |
| Bleeding (BARC definition) | 3.8% (10) | 2.4% (3) | 1.55 [0.43, 5.63] | 0.50 |
| Type 3 or Type 5 | 2.3% (6) | 0.0% (0) | — | 0.09 |

BARC = Bleeding Academic Research Consortium; DES = drug-eluting stent; DP-EES = durable polymer everolimus-eluting stents; PCI = percutaneous coronary intervention.

**Supplemental Table 6. Comparison of Outcomes Between Supreme DES and DP-EES Among Patients With Severe Tortuosity**

| **Parameter** | **Supreme DES (n=60)** | **DP-EES (n=35)** | **Hazard Ratio [95% Confidence Interval]** | **p Value** |
| --- | --- | --- | --- | --- |
| **Primary endpoint** | | | | |
| Target lesion failure | 6.8% (4) | 8.6% (3) | 0.76 [0.17, 3.39] | 0.72 |
| **Secondary endpoints** | | | | |
| Lesion success | 100% (81/81) | 100% (44/44) |  | — |
| Device success | 96.1% (73/76) | 95.5% (42/44) |  | 0.87 |
| Target vessel failure | 6.8% (4) | 8.6% (3) | 0.76 [0.17, 3.39] | 0.72 |
| Target lesion revascularization with PCI | 1.7% (1) | 0.0% (0) | — | 0.45 |
| Major adverse cardiac events | 6.8% (4) | 8.6% (3) | 0.76 [0.17, 3.39] | 0.72 |
| Stent thrombosis | 1.7% (1) | 2.9% (1) | 0.59 [0.04, 9.41] | 0.70 |
| **Components of endpoints** | | | | |
| All-cause mortality | 0.0% (0) | 2.9% (1) | — | 0.19 |
| Cardiovascular death | 0.0% (0) | 2.9% (1) | — | 0.19 |
| Myocardial infarction | 6.8% (4) | 5.7% (2) | 1.14 [0.21, 6.22] | 0.88 |
| Periprocedural | 1.7% (1) | 5.7% (2) | 0.29 [0.03, 3.22] | 0.28 |
| Non-periprocedural | 5.1% (3) | 0.0% (0) | — | 0.19 |
| Target vessel myocardial infarction | 5.0% (3) | 5.7% (2) | 0.86 [0.14, 5.14] | 0.87 |
| Periprocedural | 1.7% (1) | 5.7% (2) | 0.29 [0.03, 3.22] | 0.28 |
| Non-periprocedural | 3.3% (2) | 0.0% (0) | — | 0.28 |
| Revascularization with PCI | 3.3% (2) | 2.9% (1) | 1.14 [0.10, 12.57] | 0.92 |
| Target vessel revascularization with PCI | 3.3% (2) | 2.9% (1) | 1.14 [0.10, 12.57] | 0.92 |
| Early Stent Thrombosis (0 to 30 days after stent implantation) | 1.7% (1) | 2.9% (1) | 0.59 [0.04, 9.41] | 0.70 |
| Acute stent thrombosis (≤1 day after stent implantation) | 0.0% (0) | 0.0% (0) | — | — |
| Subacute stent thrombosis (2-30 days after stent implantation) | 1.7% (1) | 2.9% (1) | 0.59 [0.04, 9.41] | 0.70 |
| Late stent thrombosis (>30 days to 1 year after stent implantation) | 0.0% (0) | 0.0% (0) | — | — |
| **Other complications** | | | | |
| Bleeding (BARC definition) | 5.1% (3) | 2.9% (1) | 1.74 [0.18, 16.73] | 0.63 |
| Type 3 or Type 5 | 5.1% (3) | 0.0% (0) | — | 0.19 |

BARC = Bleeding Academic Research Consortium; DES = drug-eluting stent; DP-EES = durable polymer everolimus-eluting stents; PCI = percutaneous coronary intervention.

**Supplemental Table 7. Comparison of Outcomes Between Supreme DES and DP-EES Among Patients With Severe Calcification**

| **Parameter** | **Supreme DES (n=104)** | **DP EES (n=57)** | **Hazard Ratio [95% Confidence Interval]** | **p Value** |
| --- | --- | --- | --- | --- |
| **Primary endpoint** | | | | |
| Target lesion failure | 8.8% (9) | 7.0% (4) | 1.24 [0.38, 4.02] | 0.72 |
| **Secondary endpoints** | | | | |
| Lesion success | 100% (132/132) | 98.5% (64/65) |  | 0.97 |
| Device success | 92.4% (121/131) | 95.2% (60/63) |  | 0.64 |
| Target vessel failure | 10.7% (11) | 12.3% (7) | 0.86 [0.33, 2.22] | 0.75 |
| Target lesion revascularization with PCI | 4.9% (5) | 0.0% (0) | — | 0.09 |
| Major adverse cardiac events | 11.7% (12) | 14.0% (8) | 0.82 [0.34, 2.02] | 0.67 |
| Stent thrombosis | 1.9% (2) | 0.0% (0) | — | 0.29 |
| **Components of endpoints** | | | | |
| All-cause mortality | 1.0% (1) | 3.5% (2) | 0.28 [0.03, 3.10] | 0.27 |
| Cardiovascular death | 1.0% (1) | 0.0% (0) | — | 0.45 |
| Myocardial infarction | 5.8% (6) | 7.0% (4) | 0.82 [0.23, 2.91] | 0.76 |
| Periprocedural | 3.8% (4) | 7.0% (4) | 0.55 [0.14, 2.19] | 0.38 |
| Non-periprocedural | 2.9% (3) | 0.0% (0) | — | 0.20 |
| Target vessel myocardial infarction | 4.8% (5) | 7.0% (4) | 0.69 [0.18, 2.55] | 0.56 |
| Any revascularization | 7.9% (8) | 7.1% (4) | 1.12 [0.34, 3.73] | 0.85 |
| PCI | 7.9% (8) | 5.3% (3) | 1.52 [0.40, 5.71] | 0.54 |
| CABG | 0.0% (0) | 1.8% (1) | — | 0.18 |
| Target vessel revascularization with PCI | 6.9% (7) | 5.3% (3) | 1.32 [0.34, 5.12] | 0.68 |
| Early stent thrombosis (0 to 30 days after stent implantation) | 1.9% (2) | 0.0% (0) | — | 0.29 |
| Acute stent thrombosis (≤1 day) | 1.0% (1) | 0.0% (0) | — | 0.46 |
| Subacute stent thrombosis (2-30 days) | 1.0% (1) | 0.0% (0) | — | 0.46 |
| Late stent thrombosis (>30 days to 1 year after stent implantation) | 0.0% (0) | 0.0% (0) | — | — |
| **Other complications** | | | | |
| Bleeding (BARC definition) | 2.9% (3) | 10.6% (6) | 0.27 [0.07, 1.07] | 0.045 |
| Type 3 or Type 5 | 2.0% (2) | 0.0% (0) | — | 0.29 |

Values are % (n) or % (n/N). BARC = Bleeding Academic Research Consortium; CABG = coronary artery bypass grafting; PCI = percutaneous coronary intervention.
